# Extensive Longitudinal Transverse Myelitis in Systemic Lupus Erythematosus: Presentation of a Case and Literature Review

**DOI:** 10.7759/cureus.42053

**Published:** 2023-07-18

**Authors:** Rita Magola Sierra-Merlano, Óscar Iglesias-Jiménez, Paola Maria Blanco-Pertuz, Gloria Caterine Pérez-Mingan, Alberto José Sanjuanelo-Fontalvo

**Affiliations:** 1 Rheumatology, University of Cartagena, Cartagena, COL; 2 Internal Medicine, University of Cartagena, Cartagena, COL

**Keywords:** rheumatology, cyclophosphamide, autoimmune, neuropsychiatric lupus, transverse myelitis

## Abstract

Acute transverse myelitis (TM) is an inflammatory disease that manifests with motor, sensory, and autonomic symptoms of rapid progression with catastrophic outcomes; the three main causes of acute TM are demyelinating diseases, infections, and autoimmune inflammatory diseases such as systemic lupus erythematosus (SLE). TM is one of the 19 neuropsychiatric diseases associated with SLE according to the American College of Rheumatology (ACR) and has been described as affecting 1 to 2% of all cases of SLE and is frequently misdiagnosed, leading to a high rate of morbidity and mortality. This report highlights the case of a 25-year-old woman with a history of SLE who consulted for a progressive decrease in lower limb strength and loss of sphincter control, accompanied by dysesthesias from the abdomen to the feet. Upon examination, she exhibited severe paraparesis and preserved myotendinous reflexes, and a sensory level at T10 was documented. A contrast-enhanced MRI of the thoracolumbar spine was performed, showing signal hyperintensity on T2 and Short Tau Inversion Recovery (STIR) from T6 to T10. These findings are compatible with TM. Given the refractory response to initial management, the use of cyclophosphamide was required. After one week of hospital treatment, the patient achieved partial neurological recovery and was discharged for continued outpatient rheumatology care. For the diagnosis of TM in patients with SLE, a high clinical suspicion is required. Recognizing and immediately addressing this condition is crucial to prevent catastrophic outcomes and the high morbidity and mortality that stem from this association.

## Introduction

Acute transverse myelitis (TM) is an inflammatory disease that manifests with rapidly progressive motor, sensory, and autonomic symptoms and catastrophic outcomes. The three main causes of acute TM are demyelinating diseases, infections, and autoimmune inflammatory diseases such as systemic lupus erythematosus (SLE) [1]. The neurological and psychiatric manifestations of SLE (NPSLE) are varied and associated with a poor prognosis. Research using the American College of Rheumatology (ACR) NPSLE classification nomenclature reports a prevalence of 37%-95% [2]. The wide range in the prevalence of NPSLE can be explained by several factors: the inclusion of minor manifestations, the lack of a gold standard for the different syndromes, inconsistency in attributing events either to NPSLE or to secondary causes of NPSLE (such as infections or drugs), alterations in metabolism, and multi-organ damage. TM is one of the 19 less common NPSLE syndromes defined by the ACR in 1999, with a frequency of 1-2% [3]. TM has a serious clinical course; in one-third of patients, it is heraldic, but it can appear up to three years after the diagnosis of SLE. The risk of recurrence of TM is from 18 to 50% [4]. In Colombia, epidemiological records show a prevalence of SLE of 9.19 per 10,000 people, similar to other Latin American countries [5]. Myelitis affects 1-2% of SLE cases and is 1000 times more prevalent in SLE than idiopathic myelitis in the general population [3]. TM-SLE can present with the involvement of gray matter (hypotonia and hyporeflexia) and irreversible paraplegia or myelitis with white matter (spasticity and hyperreflexia). TM-SLE has high morbidity, and prompt treatment with corticosteroids and cyclophosphamide improves the prognosis; however, due to a lack of awareness of its forms of clinical presentation, TM-SLE is frequently misdiagnosed [6]. This report describes the presentation of a case of acute longitudinal TM-SLE, where clinical suspicion, rapid documentation by diagnostic images, and timely therapeutic conduct allowed full recovery from the episode. However, the loss of continuity of controls and treatment during the COVID-19 pandemic led the patient to a fatal outcome. The abstract of this article was previously presented as a poster at the XXVII ACMI conference on August 10-13, 2022.

## Case presentation

A 25-year-old woman with a history of SLE diagnosed a year ago with positive antinuclear antibody (ANA) and immune hemolytic anemia requiring transfusion, was on a treatment regimen that included prednisolone 5 mg, chloroquine 150 mg, folic acid 1 mg, and calcium with vitamin D, all for daily use. She was admitted to the ED with a one-month history of lumbar spinal pain, polyarthralgia, and progressive weakness of the lower extremities. In the past week, she reported a loss of bladder and anal sphincter control and dysesthesias extending from the abdomen to the feet. Upon evaluation in the ED, her temperature was 37°C, blood pressure was 110/70 mmHg, and heart rate was 87 beats per minute. Pulmonary, cardiac, abdominal, and articular examinations were unremarkable. The neurological examination revealed upper extremity strength of 5/5 and lower extremity strength of 1/5, reduced sensation to light touch, temperature, and pain with a sensory level in the T10 dermatome. Deep tendon reflexes were preserved, and the plantar response was bilaterally flexor. The cranial nerve evaluation did not show any abnormalities. Other findings included alopecia and sequelae of discoid cutaneous lupus. A lumbar puncture was performed, yielding clear, transparent fluid, without pleocytosis, with a glucose level of 41 mg/dl^-1^ (reference range 40-80 mg/dl^-1^) concurrent with a serum glucose level of 109 mg/dl^-1^, and total protein at 74 mg/dl^-1^ (reference range 40-60 mg/dl^-1^). Blood chemistry revealed a polymerase chain reaction (PCR) of 0.1 mg/dl-^1^ (positive > 1 mg/dl^-1^), and infectious tests in the cerebrospinal fluid (CSF) were negative. A full summary of the tests performed is presented in Table 1.

**Table 1:** Summary of laboratories and imaging study performed. AST: Aspartate Amino Transferase; ALT: Alanine Amino Transferase; CRP: C-Reactive Protein; LDH: Lactate Dehydrogenase; EBV: Epstein-Barr Virus; CMV: Cytomegalovirus; HSV-1: Herpes Simplex Virus 1; HSV-2: Herpes Simplex Virus 2; Anti-HCV: Antibodies against Hepatitis C Virus; HbsAg: Antibodies against hepatitis B surface antigen.

Diagnostic tests/imaging	Results
CBC and blood chemistry	Hemoglobin: 10.3 g/dl, Hematocrit: 31.8%, Leukocytes: 3,460, Neutrophils: 64.1%, Lymphocytes: 20.1%, Platelets: 370,000, CRP: 0.1 mg/dl, AST: 81 u/L, ALT: 61 u/L, Total Bilirubin: 0.3 mg/dL, Direct Bilirubin: 0.1 mg/dL, Glycemia: 109 mg/dL
CSF analysis	Colorless, transparent, pH: 8, RBCs: 3 cells/mm3, Leukocytes: 0/mm3, Proteins: 74 mg/dl, LDH: 24 u/dl, Glucose: 41 mg/dl.
Gram and CSF culture	Negative
CSF antibodies	EBV VCA IgG: Negative, IgM: Negative, CMV IgG: Negative, IgM: Negative, HSV1 IgG: Positive, IgM: Negative, HSV2 IgG: Negative, IgM: Negative.
Microbiology	Anti-HCV: Negative, VDRL: Non-reactive, HIV: Negative, HbsAg: Negative
other laboratories	ANA: 1/1280 homogeneous pattern, Anti-DNA: 1775 IU/ml, C3: 36 mg/dl, C4: 6.6 mg/dl, SSA/RO: 51.18 U/ml, Anti-β2 Glycoprotein (β2GPI): 6.2 U/ml, Anticardiolipin IgM: 1.02 GPL/ml, Anticardiolipin IgG: 2.6
Brain and lumbosacral CT	Without modifications.
Magnetic resonance of the spinal cord	The spinal cord from T6 to T10 shows an extensive area with a slight increase in diameter, which presents high signal intensity on T2 and STIR, with no evidence of normal contrast enhancement (Figures 1 and 2)
Other images	Electromyography/Neuroconduction: Conduction of both lower limbs with evidence of axonal compromise in the bilateral peroneal nerves and myelinated left tibial nerve is compatible with polyneuropathy.

**Figure 1:**
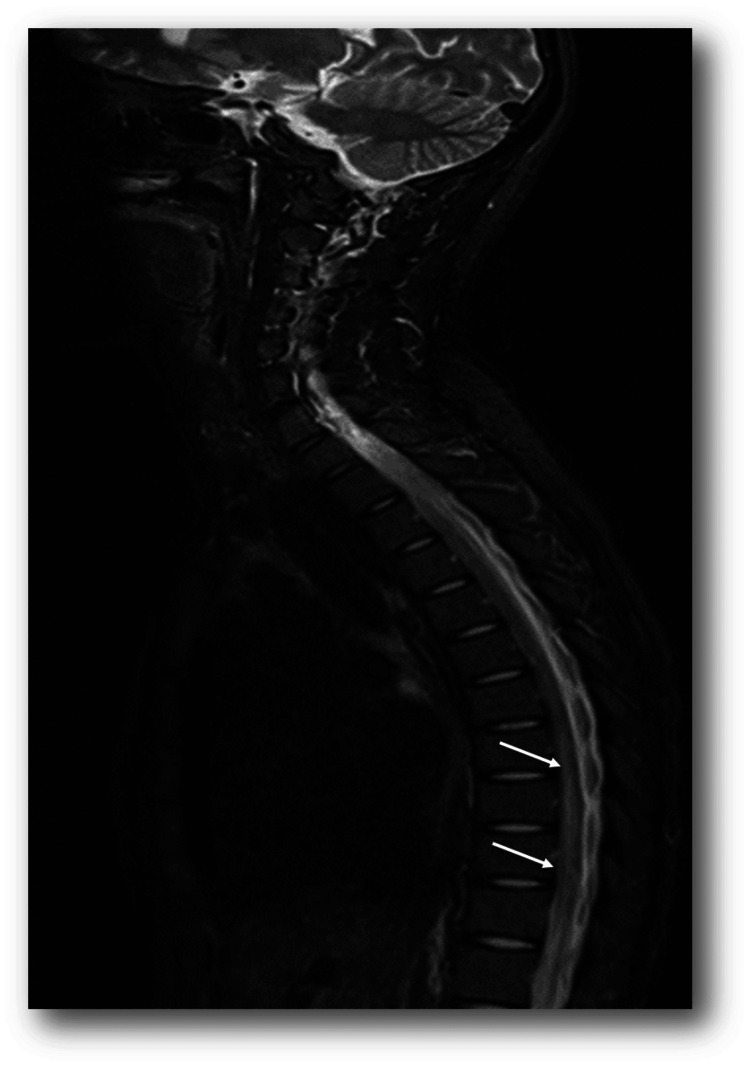
T2-weighted sagittal image revealing an extensive area with a slight increase in diameter that presents high signal intensity from the T6 to T10 level (white arrows).

**Figure 2:**
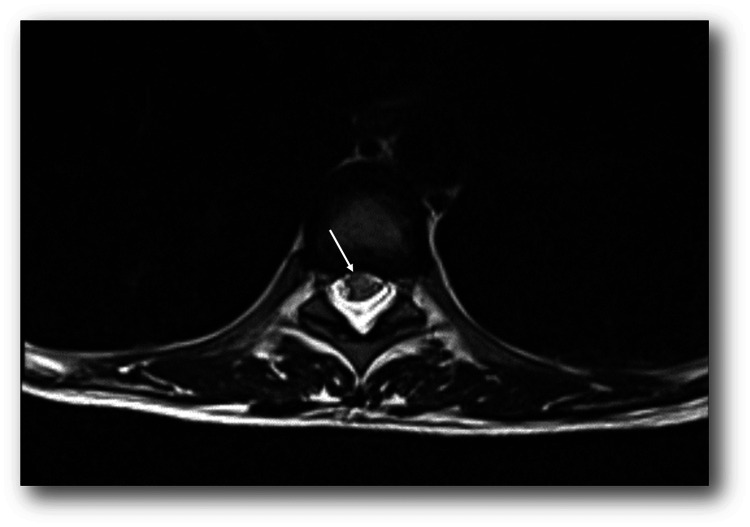
Cross-sectional T2-weighted image of the spinal cord at the T6 level showing high signal intensity (white arrow).

With suspicion of complete spinal cord syndrome, a thoracolumbar spine CT study was performed that ruled out extramedullary compression. Considering possible TM, management was started with high doses of methylprednisolone, 1 gram/day for 72 hours. New contrast-enhanced magnetic resonance images of the neuraxis were taken, showing an extensive area with a slight increase in medullary diameter, which presents signal hyperintensity on T2 and STIR from T6 to T10, consistent with the clinical findings and with the impression of longitudinally extensive TM (Figures 1-2).

## Discussion

SLE is a multisystemic disease with a complex pathophysiology, capable of affecting any organ. Its most frequent clinical forms are cutaneous and articular, but renal involvement and NPSLE are also significant. Of the latter, it has been shown that, if present, it confers greater severity and determines the prognosis of the disease [7]. NPSLE refers to neurological and psychiatric disorders derived from SLE. Its diagnosis is challenging for clinicians, given the broad spectrum and high heterogeneity of the phenotypes [8]. In relation to its categorization, in 1999, it was classified into 19 manifestations by the ACR, included in two groups: those that affect the central nervous system and are subdivided into focal and diffuse, and those that compromise the peripheral nervous system [3]. Among the focal manifestations of the central nervous system is TM. This condition goes beyond the findings of a pathological or radiological lesion in the spinal cord, as it signifies an acute or subacute spinal cord dysfunction at a sensory level. If it is complete, it also results in weakness and autonomic deterioration (bladder, intestine, and sexual) below the level of the lesion. At the same time, if it is partial, there is a motor or sensory compromise, but not both [9]. The NPSLE pathogenesis is still not fully known. Based on pathological and serological findings, it is postulated that vasculitis and small vessel thrombosis would be the two most important mechanisms responsible for axonal damage due to ischemia and necrosis. In the specific case of TM, considering the medullary level of involvement and the extent of involvement, it could be assumed which is the predominant pathophysiological mechanism. Compromise at the thoracic level (sector with the smallest caliber vessels of the medullary vasculature and therefore more vulnerable to thrombosis) is frequent, which, if associated with the presence of antiphospholipid antibodies (aPL) in serum, could indicate that thrombosis would comply a preponderant pathogenic role [10]. Case series have determined that in patients with TM due to NPSLE, the association with aPL occurs with a frequency between 50 and 100%, as in that reported by Katsiari CG et al. (2011), where no benefit was obtained from anticoagulant treatment [11]. Two relevant aspects are then generated: these antibodies indicate a greater risk of neurological manifestations in patients with SLE, and their prevalence in some cohorts seems to be higher, making it relevant to determine their positivity. A subgroup of patients with SLE is seropositive for Antiaquaporin 4 (AQP4), which is highly specific for the diagnosis of NMO and leads to direct injury to the central nervous system as a result of astrocyte injury by antibody and complement-dependent cellular cytotoxicity. The presence of AQP4 in the first episode of TM may indicate the risk of recurrence and the development of optic neuritis within less than a year [12]. While its occurrence in SLE is only 2-3%, it reaches 27% in NPSLE [13]. In the absence of local availability for its measurement, the International Consensus for Neuromyelitis Optica Spectrum Disorders proposes that, as per its most recent 2015 review, a rigorous assessment, two basic clinical criteria, and the exclusion of an alternative diagnosis are equally useful for diagnosing these disorders [14]. For the patient in the reported case, there was no documentation of spatial dissemination or involvement in multiple neuroanatomical regions. Additionally, there was no symptomatic brain syndrome or area postrema present during the onset of her myelitis. Until the time of her death, there was no evidence of new involvement in any of the six clinical regions of the CNS that are of diagnostic interest for NMO [15]. In relation to diagnosis, in 1999, the ACR established the diagnostic criteria for myelitis associated with lupus. According to the clinical presentation, patients present with acute to subacute paraparesis or quadriparesis, usually bilateral but not always symmetrical; sensory deficit localizable at a spinal sensory level; and/or impaired bowel or bladder function. Rapid neuroimaging evaluation is essential to exclude compression. Gadolinium-enhanced MRI is considered the diagnostic method of choice to confirm myelitis of any cause, including lupus. In addition, excluding other causes of spinal cord involvement other than myelitis, such as bruises or tumors, is useful. The images show a predominantly longitudinal spinal cord compromise (71%) compared to the transverse one (28%) [6]. With respect to the findings in CSF, pleocytosis may occur, which is usually lymphocytic. In addition to CSF glucose values, they may be slightly or moderately low (generally >30 mg/dl); however, a report of CSF within normal limits does not exclude the diagnosis [5]. Considering the clinical presentation and imaging findings, two subtypes of myelitis have been reported: Gray matter myelitis is characterized by fever, flaccidity, hyporeflexia, and urinary retention with a faster nadir. Its activity is associated with the presence of anti-DNA. White matter myelitis is associated with positive aPL and anti-Ro/SSA with recurrent thrombosis [16]. In the cohort studied by Birnbaum J et al. in 2009, it was found that in MT-SLE overlapping with NMO, white matter involvement is preponderant, with a common history of optic neuritis, longitudinally extensive myelitis (>3 vertebral segments), presentation with periodic relapses, and absence of multiple sclerosis imaging criteria, on the other hand, when the involvement involves the substance gray shows a monophasic pattern of recurrence. In a subgroup of patients with antiphospholipid syndrome (APS), white matter involvement with positive lupus anticoagulant predominates in more than half of the patients, contrasting with gray matter involvement, where it barely reaches 20%. When anti-DNA and anti-RO/SSA antibodies were evaluated, there was no statistical difference between these groups [6]. The case described did not meet the criteria for NMO or APS, and its involvement, despite presenting mixed characteristics, had a predominance of gray matter involvement (Table 2).

**Table 2:** Comparison between white and gray matter disease in MT-SLE. Gd: Gadolinium; CSF: Cerebrospinal fluid; SLE: Systemic Lupus Erythematosus; LETM: Longitudinally extensive transverse myelitis; NMO: Neuromyelitis optica; ↑­ frequent; ­­↑↑ very frequent; ↓ decreased; - absent.

	White matter	Gray matter	Informed case
Presentation	Upper motor neuron	Lower motor neuron	Lower motor neuron
Prodrome	­↑	­↑↑	-
Long-term disability	­↑	­↑↑	­↑
CSF	Leukocytes:↑ Proteins:↑ Glucose: Normal	Leukocytes:↑↑ Proteins: ↑↑ Glucose: ↓	Leukocytes:- Proteins: ↑ Glucose: Normal
MRI	LETM: ↑↑ Gd enhacement ↑↑	LETM: ↑ Gd enhacement ↑	LETM: ↑↑ Gd enhacement -
Recurrence	­↑↑	-	-
Optic neuritis	­↑	-	-
SLE activity	­↑	-	-

In relation to treatment, the European League Against Rheumatism (EULAR) recommends, in its guidelines for neuropsychiatric manifestations in lupus, an early initiation of methylprednisolone and intravenous (IV) cyclophosphamide treatment (grade A recommendation). This should ideally begin within the first hours of symptom onset, even in cases where the cerebrospinal fluid (CSF) suggests meningitis, while microbiological studies are being performed. The combination of these medications is considered the standard therapy for this neuropsychiatric complication [17]. The doses used are 1 gram pulses of methylprednisolone intravenously for three days together with IV cyclophosphamide at 0.75 -1 g/m2 of body surface area monthly for six months to a year and then every three months for a year, together with oral prednisone 1 mg/kg/day on the fourth day of starting treatment, with a gradual decrease after 1-3 months. In refractory cases, plasmapheresis can be added between 6 and 10 sessions, although it does not seem to improve the prognosis [18]. Intravenous gammaglobulin has also been used as initial therapy or, in refractory cases, alone or accompanying standard treatment. Although anticoagulation is used in cases of aPL-positive myelitis, as mentioned above, it has not shown any additional therapeutic benefit to immunosuppression [19]. Current data regarding the use of biologics such as rituximab alone or in combination with cyclophosphamide seem to show promising results, but larger methodological studies are needed [20].

Some therapeutic options in the maintenance phase include azathioprine, methotrexate, or mycophenolate in conjunction with low-dose steroids. It is usually recommended for three or more years; however, the optimal duration remains to be defined.

## Conclusions

SLE can occasionally cause a serious neurological consequence called longitudinal extensive transverse myelitis (LETM). Literature significantly lacks information on the clinical course, outcome, and therapeutic effectiveness. Infections, demyelinating conditions, and compressive tumors are all included in the broad differential diagnosis of LETM. Due to the clinical and prognostic importance of MT-SLE, immediate recognition and intervention are required to prevent catastrophic outcomes that occur in up to one-third of patients. If clinical improvement is not achieved with the initial corticosteroid therapy, the addition of immunomodulatory management that includes cyclophosphamide or plasmapheresis should not be delayed due to its favorable impact on disability and survival, especially in refractory cases. Our patient had LETM and displayed substantial neurologic deterioration. An opportune intervention brought about a remarkable gradual recovery of function, and our SLE patient could walk and recover sphincter function, and sensation, without presenting long-term sequelae.
